# Impact of Rotaviral Diarrhea on Child Growth in Sub-Saharan Africa and South Asia in the Global Enteric Multicenter Study

**DOI:** 10.4269/ajtmh.23-0406

**Published:** 2024-02-20

**Authors:** Rukaeya Amin Sobi, Al-Afroza Sultana, Soroar Hossain Khan, Md. Ahshanul Haque, Sharika Nuzhat, Md. Nasif Hossain, Pradip K. Bardhan, Mohammod Jobayer Chisti, Subhra Chakraborty, Tahmeed Ahmed, Rina Das, Abu S. G. Faruque

**Affiliations:** ^1^ Nutrition Research Division, International Center for Diarrheal Disease Research, Bangladesh (icddr,b), Dhaka, Bangladesh;; ^2^ James P. Grant School of Public Health, BRAC University, Dhaka, Bangladesh;; ^3^ Department of Global Health, School of Tropical Medicine and Global Health, Nagasaki University, Nagasaki, Japan;; ^4^ Johns Hopkins Bloomberg School of Public Health, Johns Hopkins University, Baltimore, Maryland;; ^5^ Department of Global Health, University of Washington, Seattle, Washington;; ^6^ Gangarosa Department of Environmental Health, Rollins School of Public Health, Emory University, Atlanta, Georgia

## Abstract

Rotavirus is the leading cause of dehydrating diarrhea among children in developing countries. The impact of rotaviral diarrhea on nutritional status is not well understood. We aimed to determine the association between rotavirus-positive moderate-to-severe diarrhea and nutrition in children under 5 years of age. We analyzed data from the Global Enteric Multicenter Study on children 0–59 months old from South Asia and sub-Saharan Africa. The relationships between explanatory variables and outcome variables were assessed using multiple linear regression; the explanatory variable was the presence of rotavirus in the stool sample, and the outcome variables were *z* scores [length/height-for-age (LAZ/HAZ), weight-for-age (WAZ), and weight-for-length/height (WLZ/WHZ)] at follow-up (∼60 days). The prevalence of rotaviral diarrhea was 17.3% (905/5,219) in South Asia and 19.95% (842/4,220) in sub-Saharan Africa. Rotavirus was associated with higher LAZ/HAZ (β: 0.19; 95% CI: 0.12, 0.26; *P* <0.001) and WAZ (β: 0.15; 95% CI: 0.79, 0.22; *P* <0.001) in sub-Saharan Africa and with lower WLZ/WHZ (β coefficient: −0.08; 95% CI: −0.15, −0.009; *P* = 0.027) in South Asia. Our study indicates that rotaviral diarrhea is positively associated with nutritional status in sub-Saharan Africa and is negatively associated with nutritional status in South Asia. An expedited implementation policy of ongoing preventive and control strategies, including vaccination against rotavirus, is necessary to reduce the burden of rotaviral diarrhea, which may further help to reduce the potential nutritional ramifications.

## INTRODUCTION

Globally, diarrhea imposes a significant role in mortality and morbidity^1^ and accounts for 9% of all deaths in children under 5 years old.^2^ Despite the increasing availability of simple treatment solutions such as oral rehydration solution (ORS), approximately 484,000 children die each year as a result of diarrhea.^2^ Diarrhea has a diverse etiology.^3^ Rotavirus is one of the most common causes of moderate-to-severe diarrhea (MSD)^3^ and is estimated to cause approximately 185,000 deaths/year worldwide among children under 5 years.^4^ The number of annual diarrheal episodes and deaths among children under 5 years is significantly higher in Southeast Asia and sub-Saharan Africa than in developed countries.^5^ Moreover, the Global Enteric Multicenter Study (GEMS) reported rotavirus as a leading cause of severe diarrhea among children across sub-Saharan Africa and South Asia.^3^ Rotavirus vaccination has been included in widely implemented national immunization programs in developing countries. However, despite the introduction of rotavirus vaccination in several developing countries, around 260 million diarrheal diseases occur annually among children under 5 years, and >1.5 million cases require hospitalization.^6^

Various factors are associated with rotavirus infection, including nutritional status, dehydration, episode occurring in the winter, dry season, and age under 2 years.^7^^–^^10^ Moreover, rotavirus-positive diarrhea often becomes more serious among malnourished children.^9^^,^^11^ Several field-based^12^^,^^13^ and animal studies^14^^–^^17^ have shown that malnutrition affects the immune system, alters innate and adaptive immune responses, impairs epithelial cell barrier integrity, and leads to dysfunction within intestinal epithelial cells. A study in Burkina Faso reported rotavirus as the leading etiology of pediatric diarrhea (32.4%), and the clinical severity score of children with rotavirus-positive diarrhea was associated with childhood malnutrition.^18^ Another study from Mozambique observed that around 27.2% of undernourished children with diarrhea were rotavirus-positive.^19^ However, counter to this evidence that malnutrition can weaken the immune system and result in a higher incidence of common childhood illnesses,^20^ shreds of evidence from several studies have also suggested a protective relationship between malnutrition and rotaviral diarrhea.^20^^–^^23^ Two different cross-sectional studies conducted in Bangladesh found that better nutritional status was associated with a significantly higher incidence of rotaviral diarrheal episodes among children under 5 years.^22^^,^^23^ However, studies in South Asia and sub-Saharan Africa have also indicated that the burden and effects of rotavirus may manifest differently in these geographic regions. Thus, given the conflicting findings between different studies in different regions of the world, the relationship between rotaviral diarrhea and childhood malnutrition is still controversial.

As described above, investigations of rotaviral diarrhea and its influence on child growth have revealed varied significant associations in specific regions, cities, and countries.^18^^,^^19^ To address this issue, we expanded this analysis to a broader scope by utilizing an identical standardized methodology to explore the association between rotaviral (+) MSD and subsequent child growth in multiple settings of sub-Saharan Africa and South Asia using data from the GEMS study.

## MATERIALS AND METHODS

### The GEMS study design and participants.

The GEMS was a prospective, age-stratified, matched case-control study conducted over 36 months from December 2007 to February 2011 in seven study sites across sub-Saharan Africa (The Gambia, Mali, Mozambique, and Kenya) and South Asia (Bangladesh, India, and Pakistan).^24^ Children under 5 years of age from the Demographic Surveillance System catchment area who presented to the Sentinel Health Center with MSD and who were hospitalized within 7 days of the onset of acute illness were considered cases.^24^ Nutritional assessments based on weight, length/height, and mid-upper arm circumference were performed at the time of enrollment.^24^ The GEMS field workers visited the household of each enrolled child around 60 days after enrollment (acceptable range, 50–90 days).^24^ Nutritional measurement and details of comorbidities (malaria, typhoid, pneumonia, diarrhea, and dysentery) within the ∼60-day follow-up period were collected from the follow-up household visits for analysis. Details of the GEMS have previously been described in detail.^24^

**Figure 1.**
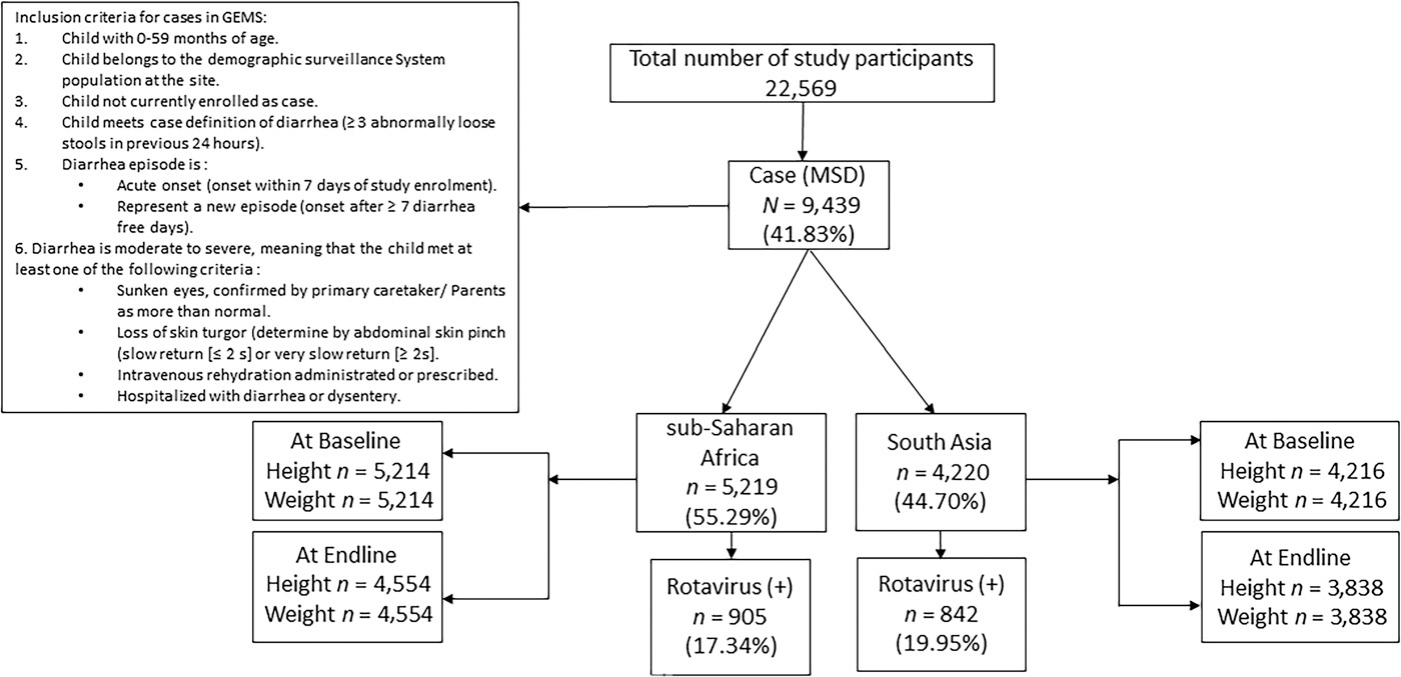
Study flowchart of the inclusion of children under 5 years of age with rotavirus-positive moderate-to-severe diarrhea in sub-Saharan Africa and South Asia. GEMS = Global Enteric Multicenter Study; MSD = moderate-to-severe diarrhea; Rotavirus (+) = rotavirus-positive.

In the current analysis, we included children under 5 years of age with rotavirus-positive MSD who were enrolled in the GEMS, including 905 (17.34%) rotavirus-positive cases from sub-Saharan Africa and 842 (19.95%) from South Asia (Figure 1). Data were also extracted on children under 5 years with rotavirus-negative MSD who were enrolled in the GEMS as a comparison group for some analyses. Length/height-for-age (LAZ/HAZ), weight-for-age (WAZ), and weight-for-length/height (WLZ/WHZ) were determined at baseline and end line for all children.

For MSD, the episodes of diarrhea had to be acute (onset within 7 days of enrollment) and current (onset after ≥7 diarrhea-free days), and at least one of the following characteristics had to be met: sunken eye; loss of skin turgor (abdominal skin pinch with slow return [≤2 seconds] means some dehydration and or very slow return [>2 seconds] means severe dehydration); intravenous hydration administrated or prescribed; dysentery (visible blood in stool); or admission to the hospital with diarrhea or dysentery.^25^

Figure 1 shows the study flowchart of the inclusion of children under 5 years with rotavirus-positive MDS in sub-Saharan Africa and South Asia.

#### Fecal microbiology and rotavirus identification.

Fresh stool samples for the GEMS were collected at the time of enrollment according to the protocol developed for the GEMS laboratory procedures protocol.^26^ The GEMS protocol used standard laboratory methods to identify bacterial, viral, and protozoal pathogens. All protocols were adapted from the Manual of Clinical Microbiology, eighth edition.^26^^,^^27^ Virus immunoassays were performed using ELISA, rapid, robust, sensitive, and specific diagnostic assays that are widely used to detect viral pathogens. The ProSpecT ELISA (Oxoid, Basingstoke, United Kingdom) rotavirus kit, a well-established commercial immunoassay, was used to identify rotavirus VP6 antigen in stool.^26^

### Outcome variables.

In this analysis, the primary measures of growth were LAZ/HAZ, WAZ, and WLZ/WHZ (at enrollment and follow-up) calculated according to WHO guidelines.^24^

### Variables of interest.

Diarrhea was described as passage of three or more abnormally loose or watery stools per day, and acute diarrhea was defined as suffering diarrhea for 1–6 days within the previous 21 days. Other variables such as vomiting (at least three times per day), fever (measuring at least 38°C), and required intravenous (IV) rehydration were also recorded.^24^ Children who were currently either exclusively or partially breastfeeding were considered “breastfed.” Information on each study participant’s household (defined as a group of people who share a common cooking fire) included the mother as a primary caregiver, mother’s educational status (literate or illiterate), handwashing practice (before nursing or preparing the child’s food, cleaning a child after defecation), use of handwashing materials (water with soap or without soap), source of drinking water (tube well water and non-tube well water), method of treatment of drinking water, and presence of improved sanitation facilities (toilet facility available for disposal of human fecal material or not). All these variables were taken into consideration as explanatory variables in this analysis. In the assessment of factors associated with disease, household socioeconomic status (SES) was categorized based on household income into wealth index quintiles (poor, lower-middle, middle, upper-middle, and richest) as previously described.^24^

### Anthropometry.

For each enrolled child, length/height, weight, and mid-upper arm circumference were measured during enrollment (baseline) and at the ∼60-day follow-up visit (end line), as previously described in detail.^25^ Using the WHO Child Growth Standards as the reference population, the LAZ/HAZ, WAZ, and WLZ/WHZ were calculated using the WHO Child Growth Standards as the reference population, with a WHO statistical analysis software macro.^28^^,^^29^ According to WHO 2008 guidelines, the children were defined as stunted if LAZ/HAZ was <−2 SDs, underweight by having WAZ <−2 SDs, and wasted as having WLH/WHZ< −2 SDs.^30^

### Statistical analyses.

The child, mother, and household were summarized as mean and SD for continuous variables and as frequency and percentage for categorical variables. We performed multiple logistic regression analyses to explore the factors associated with rotavirus infection. Paired *t*-tests were conducted to assess the differences in *z* scores between baseline (at enrollment) and end line (60–90 days’ follow-up after enrollment) among the study participants (both rotavirus positive and negative). A generalized linear model was used to individually investigate the adjusted impact of the explanatory variable (presence of fecal rotavirus) on the outcome variables (WAZ, WLH/WHZ, and LAZ/HAZ). In multivariable modeling of the association of rotaviral (+) MSD with explanatory variables, all the covariates, including age, breastfeeding status, clinical features, mother’s level of education, handwashing before nursing and after cleaning a child, handwashing practice, the main source of drinking water, wealth index, available sanitation facilities, co-pathogens frequently isolated in the stool (*Cryptosporidium*,* Campylobacter*,* Giardia*, enteroaggregative *Escherichia coli* [EAEC], enterotoxigenic *E. coli* [ETEC], time (anthropometry at two time points: on enrollment and day 60 follow-up), and study site, were selected based on a literature review. The Mantel-Haenszel test was used to explore the association of rotavirus-positive MSD with changes in child anthropometry within different regions, and the likelihood-ratio test was performed to test the rotaviral diarrhea sites’ interaction using a logistic model; we did not observe any effect modification for HAZ, WAZ, and WHZ. To describe the precision of point estimates, we calculated the β coefficient and 95% CI. A variable with a *P*-value <0.05 was considered statistically significant in all analyses. STATA 15.0 IC (Stata Crop LLC, College Station, TX) was used to analyze the data.

## RESULTS

### General characteristics of the study population with rotavirus-positive MSD.

During the GEMS, a total of 1,747 rotavirus-positive stool samples were obtained from 9,439 enrolled participants under 5 years old with symptomatic MSD who completed 60 days of follow-up. The baseline demographic characteristics of the children with rotavirus-positive MSD are presented in Table 1.

**Table 1:** Baseline characteristics of the children under 5 years of age with rotavirus-positive moderate-to-severe diarrhea

Characteristics	Sub-Saharan Africa	South Asia
	*N* = 5,219 (%)	*N* = 4,220 (%)
Rotavirus-positive (*N* = 1,747)	905 (17.3)	842 (19.9)
Age (months)*	11.84 ± 8.7	12.68 ± 8.7
Age group		
0–11 months	544 (60.1)	472 (56.0)
12–23 months	282 (31.1)	290 (34.4)
24–59 months	79 (8.7)	80 (9.5)
Sex (female)	431 (47.6)	373 (44.3)
Currently breastfed (yes)	779 (86.0)	742 (88.1)
Baseline anthropometry		
LAZ/HAZ*	−0.95 ± 1.4	−1.33 ± 1.3
WAZ*	−1.27 ± 1.4	−1.62 ± 1.3
WLZ/WHZ*	−0.99 ± 1.5	−1.18 ± 1.5
Clinical features		
Required intravenous rehydration	280 (30.9)	172 (20.4)
Fever^†^	181 (20.0)	123 (14.6)
Vomiting ≥3 times/day	650 (71.8)	508 (60.3)
Sociodemographic characteristics		
Mother’s education (literate)	458 (50.9)	520 (61.9)
SES		
Poor	166 (18.4)	214 (25.4)
Lower middle	167 (18.5)	143 (16.9)
Middle	205 (22.5)	154 (18.3)
Upper middle	180 (19.9)	169 (20.1)
Richest	186 (20.6)	162 (19.2)
WASH		
Main source of drinking water		
Tube well	54 (5.9)	221 (26.2)
Non-tube well	851 (94.0)	621 (73.7)
Use of water treatment method		
Yes	229 (25.3)	316 (37.5)
No	676 (74.7)	526 (62.5)
Improved toilet facility available		
Yes	854 (94.4)	821 (97.5)
No	51 (5.64)	21 (2.49)
Handwashing practice		
Water with soap	729 (80.6)	575 (68.3)
Water without soap	175 (19.4)	267 (31.7)
Handwashing before nursing a child or preparing baby’s food		
Yes	303 (33.5)	478 (56.8)
No	602 (66.5)	364 (43.2)
Handwashing after cleaning children who have defecated		
Yes	349 (38.6)	440 (52.3)
No	556 (61.4)	402 (47.7)
Co-pathogens isolated in stool		
* Cryptosporidium*	53 (5.9)	108 (12.8)
* Campylobacter*	59 (6.5)	109 (12.9)
* Giardia*	119 (13.1)	89 (10.6)
EAEC	205 (22.6)	181 (21.5)
ETEC	93 (10.3)	52 (6.2)
* Shigella*	18 (1.9)	33 (3.9)

EAEC = enteroaggregative *Escherichia coli*; ETEC = enterotoxigenic *Escherichia coli*; LAZ/HAZ = length/height-for-age z-score; MSD = moderate-to-severe diarrhea; SES = socioeconomic status; WASH = water, sanitation, and hygiene; WAZ = weight-for-age z score; WLZ/WHZ = weight-for-length/height z-score. The reference groups for co-pathogens are the absence of that specific pathogen.

* Mean ± SD.

^†^ Fever: measured at least 38°C.

The mean age of the children with rotavirus-positive MSD was approximately 12 months in both regions. In sub-Saharan Africa and South Asia, 60.1% and 56.0% of children with rotavirus-positive MSD were between 0 and 11 months old and 47.6% and 44.3% were female, respectively. More than 85% of children with rotavirus-positive MSD were currently breastfed. Higher proportions of children with rotavirus-positive MSD required IV rehydration (30.9% versus 20.4%) and presented with fever (20.0% versus 14.6%) and vomiting (71.8% versus 60.3%) in sub-Saharan Africa than in South Asia. The mean and SD LAZ/HAZ, WAZ, and WLZ/WHZ of the children with rotavirus-positive MSD at enrollment were −0.95 ± 1.4, −1.27 ± 1.4, −0.99 ± 1.5, respectively, in sub-Saharan Africa and −1.33 ± 1.3, −1.33 ± 1.3, −1.18 ± 1.5, respectively, in South Asia; all three anthropometric indices were lower in South Asia than in sub-Saharan Africa.

Mothers were considered the main caregivers of children with rotavirus-positive MSD in both regions, and >50% of caregivers were literate. There were no notable differences in the distributions of the households of the children with rotavirus-positive MSD across the five SES categories between the two regions. More than 90% of the respondents’ households had improved toilet facilities in both sub-Saharan Africa and South Asia. Non-tube wells were the main source of drinking water in both regions; however, a higher percentage of households used non-tube well water in sub-Saharan Africa than in South Asia (94.0% versus 73.7%). Most of the households in both regions did not use any water treatment methods. Almost 80% of respondents used soap and water during handwashing in sub-Saharan Africa compared with 68.3% of respondents in South Asia. However, a higher percentage of respondents reported handwashing before nursing or preparing food for children (56.8% versus 33.5%) and cleaning the children after defecation (52.3% versus 38.6%) in South Asia than in sub-Saharan Africa.

In the analysis of stool samples from children with rotavirus-positive MSD, *Cryptosporidium, Campylobacter, Giardia*, EAEC, and ETEC were detected as co-pathogens in both regions (Table 1); EAEC was reported as a leading co-pathogen in both sub-Saharan Africa (22.65%) and South Asia (21.50%).

### Baseline factors associated with rotavirus-positive MSD.

Multivariate analysis (Table 2) of the clinicopathological, household, and socioeconomic factors associated with rotavirus-positive MSD at baseline revealed that younger age (0–11 months) was associated with a higher risk of rotavirus-positive MSD than the other age categories overall; however, this association was significant in South Asia but not in sub-Saharan Africa. Female sex was associated with a higher risk of rotavirus-positive MSD overall compared with male sex; however, this association was only significant in sub-Saharan Africa. In terms of clinical features, rotavirus-positive MSD was associated with the presence of fever in South Asia and with the absence of fever in sub-Saharan Africa. In addition, rotavirus-positive MSD was associated with a high risk of requiring IV rehydration overall; this risk was more significant in sub-Saharan Africa than in South Asia. The presence of rotavirus in MSD cases was strongly associated with frequent vomiting overall as well as in both region. Current breastfeeding was not associated with the risk of rotavirus-positive MSD overall or in either region compared with non-current breastfeeding status shows any protective role for rotavirus infection.

**Table 2:** Multiple logistic regression analysis of the factors associated with rotavirus-positive moderate-to-severe diarrhea among children under 5 years of age from sub-Saharan Africa and South Asia

Factors	Sub-Saharan Africa		South Asia		Overall	
	aOR* (95% CI)	*P*-Value	aOR* (95% CI)	*P*-value	aOR* (95% CI)	*P*-Value
Age group						
0–11 months	Ref	–	Ref	–	Ref	–
12–23 months	0.80 (0.59–1.08)	0.147	0.80 (0.68–0.94)	0.007	0.80 (0.68–0.95)	0.010
24–59 months	0.52 (0.23–1.17)	0.118	0.36 (0.24–0.55)	<0.001	0.45 (0.32–0.64)	<0.001
Sex						
Boy	Ref	–	Ref	–	Ref	–
Girl	1.33 (1.04–1.69)	0.020	1.03 (0.88–1.20)	0.676	1.18 (1.00–1.40)	0.045
Stunting						
No	Ref	–	Ref	–	Ref	–
Yes	1.09 (0.99–1.21)	0.063	1.07 (0.99–1.15)	0.051	1.07 (1.02–1.11)	0.001
Currently breastfed						
No	Ref		Ref	–	Ref	
Yes	2.08 (1.42–3.04)	<0.001	1.84 (1.39–2.45)	<0.001	1.92 (1.64–2.24)	<0.001
Clinical features						
Fever						
No	Ref	–	Ref	–	Ref	–
Yes	0.72 (0.62–0.82)	<0.001	1.22 (1.05–1.42)	0.009	0.81 (0.69–0.96)	0.015
Required intravenous rehydration						
No	Ref	–	Ref	–	Ref	–
Yes	1.65 (1.38–1.98)	<0.001	2.38 (0.69–8.18)	0.168	1.74 (1.19–2.55)	0.004
Vomiting						
No	Ref	–	Ref	–	Ref	–
Yes	3.24 (2.22–4.75)	<0.001	3.53 (2.28–5.49)	<0.001	3.33 (2.58–4.30)	<0.001
Sociodemographic characteristic						
Mother’s education						
Literate	Ref	–	Ref	–	Ref	Ref
Illiterate	1.08 (0.86–1.35)	0.520	1.02 (0.88–1.19)	0.784	1.06 (0.84–1.33)	0.596
SES						
Poorest	Ref	–	Ref	–	Ref	Ref
Lower middle	0.95 (0.56–1.62)	0.866	0.88 (0.73–1.05)	0.165	0.89 (0.69–1.14)	0.362
Middle	1.19 (1.01–1.39)	0.030	0.81 (0.65–1.01)	0.069	0.96 (0.74–1.24)	0.766
Upper middle	1.14 (0.95–1.37)	0.150	0.99 (0.97–1.00)	0.204	1.03 (0.90–1.17)	0.642
Richest	1.06 (0.93–1.19)	0.374	0.96 (0.86–1.09)	0.562	0.96 (0.86–1.08)	0.543
WASH						
Main source of drinking water						
Tube well	Ref	–	Ref	–	Ref	Ref
Non-tube well	1.02 (0.85–1.24)	0.778	1.69 (1.05–2.73)	0.030	1.04 (0.78–1.39)	0.782
Improved toilet facility available						
Yes	Ref	–	Ref	–	Ref	Ref
No	0.98 (0.79–1.20)	0.829	0.73 (0.51–1.03)	0.081	0.83 (0.64–1.08)	0.160
Handwashing practice						
Water with soap	Ref	–	Ref	–	Ref	Ref
Water without soap	0.97 (0.73–1.28)	0.852	0.92 (0.75–1.13)	0.436	1.03 (0.91–1.16)	0.641
Handwashing before nursing a child						
No	Ref	–	Ref	–	Ref	Ref
Yes	1.32 (1.02–1.73)	0.037	0.98 (0.75–1.28)	0.896	1.30 (1.01–1.69)	0.041
Handwashing after cleaning a child who defecated						
No	Ref	–	Ref	–	Ref	Ref
Yes	1.05 (0.76–1.45)	0.750	0.78 (0.74–0.82)	<0.001	0.99 (0.86–1.13)	0.847
Co-pathogens isolated in stool						
* Campylobacter*	0.83 (0.77–0.90)	<0.001	0.62 (0.45–0.82)	0.003	0.74 (0.59–0.92)	0.006
* Giardia*	0.76 (0.60–0.96)	0.024	0.72 (0.46–0.85)	0.044	0.73 (0.63–0.85)	<0.001
ETEC	0.68 (0.57–0.82)	<0.001	0.53 (0.31–0.91)	0.021	0.61 (0.48–0.78)	<0.001
EAEC	1.01 (0.92–1.11)	0.808	0.99 (0.93–1.06)	0.814	0.99 (0.94–1.05)	0.921

aOR = adjusted odds ratio; EAEC = enteroaggregative *Escherichia coli*; ETEC = enterotoxigenic *E. coli*; SES = socioeconomic status; stunting: length/height-for-age *z*-score ≤2 (%; for <5 years of age); Ref = Reference; WASH = water, sanitation, and hygiene.

* Adjusted for age, sex, clinical features, sociodemographic characteristics, WASH, wealth index, presence of co-pathogens, and site as a cluster; variables with a *P*-value of 0.05 were considered for inclusion in the final model.

Regarding SES, in sub-Saharan Africa, children from households with middle-class SES were significantly more likely to be affected by rotavirus-positive MSD than children from other SES categories; however, there were no significant associations between rotavirus-positive MSD and the other SES categories overall or in South Asia. Symptomatic rotavirus-positive MSD was more closely associated with household use of non-tube well water than with use of tube well water in South Asia, but the relation was not significant overall or in sub-saharan Africa. Respondents’ handwashing with soap was not associated with rotavirus-positive MSD overall in either region compared with handwashing without soap. Handwashing before nursing children or preparing children’s food was associated with a higher risk of rotavirus-positive MSD in sub-Saharan Africa and overall, but this relationship was not significant in South Asia. Handwashing after cleaning children who had defecated was associated with a lower risk of rotavirus-positive MSD in South Asia compared with not handwashing, though this association was not significant overall or in sub-Saharan Africa.

Finally, the co-pathogens *Campylobacter, Giardia,* and ETEC were all individually significantly associated with lower risk of rotavirus-positive MSD overall and in both regions compared with the absence of these co-pathogens in stool (Table 2).

### Changes in *Z*-scores between baseline and end line in children with rotavirus-positive MDS.

**Figure 2.**
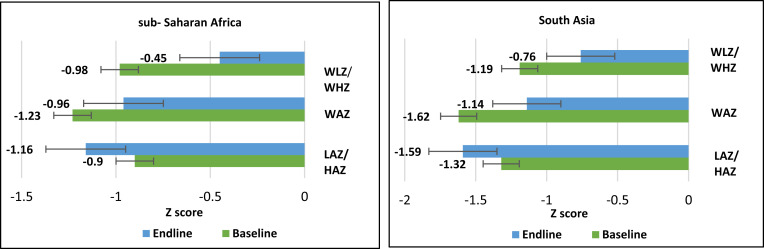
Mean baseline and end line length/height-for-age *z*-score (LAZ/HAZ), weight-for-age *z*-score (WAZ), and weight-for-length/height *z*-score (WLZ/WHZ) of the children under 5 years of age with rotavirus-positive moderate-to-severe diarrhea from sub-Saharan Africa and South Asia. The Error bar illustrates the changes in the nutritional status of children from South Asia and Sub-Saharan Africa who participated in the GEMS study. The findings, encompassing both baseline and endline assessments, reveal a noteworthy decline in the mean Height-for-Age z-score across both regions. However, the mean WHZ and WAZ increased in both regions, indicating an improvement in both Weight-for-Height and Weight-for-Age z-scores when compared to the period marked by diarrhoeal episodes.

Among children under 5 years old with rotavirus-positive MSD, mean WLZ/WHZ score and WAZ increased between baseline and end line (∼60 days) in both regions; however, LAZ/HAZ decreased between baseline and end line in both sub-Saharan Africa and South Asia (Figure 2). Similar anthropometric trends were observed in children with rotavirus-negative MSD from the GEMS study (Supplemental Material).

### Associations between rotavirus-positive MSD and changes in children’s HAZ/LAZ, WAZ, and WHZ/WLZ.

Multiple linear regression was performed to assess the association between rotavirus-positive MSD and changes in the children’s LAZ/HAZ, WAZ, and WLZ-WHZ between baseline and end line as dependent variables (Table 3). After adjustment for all potential covariates, significant associations were observed between rotavirus-positive MSD and change in all three outcomes overall: Significant positive associations were observed between rotavirus-positive MSD and changes in LAZ/HAZ (adjusted coefficient: 0.13; 95% CI: 0.08, 0.18; *P*-value: <0.001) and change in WAZ (adjusted coefficient: 0.08; 95% CI: 0.02, 0.13; *P*-value: <0.003), and a significant negative association was observed between rotavirus-positive MSD and change in WLZ/WHZ (adjusted coefficient: −0.19; 95% CI: −0.23, −0.14, *P*-value: <0.001). In sub-Saharan Africa, rotavirus-positive MSD was positively associated with change in LAZ/HAZ (adjusted coefficient: 0.19; 95% CI: 0.12, 0.26; *P*-value: <0.001) and change in WAZ (adjusted coefficient: 0.15; 95% CI: 0.09, 0.22; *P*-value: <0.001), but was not significantly associated with change in WLZ/WHZ. In South Asia, the association between rotavirus-positive MSD and changes in LAZ/HAZ and WAZ were not significant; however, a significant negative association was observed between rotavirus-positive MSD and change in WLZ/WHZ (adjusted coefficient: −0.08; 95% CI: −0.15, −0.009; *P*-value: 0.027).

**Table 3:** Multiple linear regression modeling of the associations between rotavirus-positive MSD and the dependent variables changes in child’s LAZ/HAZ, WAZ, and WLZ/WHZ between baseline and end line

*Z*-score	Overall				Sub-Saharan Africa				South Asia			
	Unadjusted Coef (95% CI)	*P *Value*	Adjusted Coef* (95% CI)	*P*-Value*	Unadjusted Coef (95% CI)	*P*-Value*	Adjusted Coef* (95% CI)	*P*-Value*	Unadjusted Coef (95% CI)	*P*-Value*	Adjusted Coef* (95% CI)	*P*-Value*
LAZ/HAZ	0.12 (0.08 to 0.16)	<0.001	0.13 (0.08 to 0.18)	<0.001	0.26 (0.19 to 0.33)	<0.001	0.19 (0.12 to 0.26)	<0.001	0.17 (0.09 to 0.24)	<0.001	0.06 (0.007 to 0.13)	0.08
WAZ	−0.01 (−0.05 to 0.03)	0.483	0.08 (0.02 to 0.13)	0.003	0.17 (0.10 to 0.24)	<0.001	0.15 (0.09 to 0.22)	<0.001	0.11 (0.04 to 0.19)	0.002	−0.009 (−0.08 to 0.06)	0.782
WLZ/WHZ	−0.1 (−0.14 to −0.05)	<0.001	−0.19 (−0.23 to −0.14)	<0.001	0.07 (−0.01 to 0.15)	0.087	0.06 (−0.02 to 0.14)	0.118	0.03 (−0.04 to 0.11)	0.347	−0.08 (−0.15 to −0.009)	0.027

Coef = coefficient; EAEC = enteroaggregative *Escherichia coli*; ETEC = enterotoxigenic *E. coli*; LAZ/HAZ = length/height-for-age *z*-score; MSD = moderate-to-severe diarrhea; WAZ = weight-for-age *z*-score; WLZ/WHZ = weight-for-length/height *z*-score.

* Adjusted for age group, gender, clinical features, mother’s education, breastfeeding, baseline anthropometry, the main source of drinking water, handwashing material, available toilet facility, wealth index, co-pathogens (*Campylobacter, Giardia*, ETEC, EAEC), and site.

## DISCUSSION

This study was designed to assess the effects of rotavirus-positive MSD on anthropometric indices and explore associations between rotavirus-positive MSD and clinicopathological, household, and socioeconomic factors among children under 5 years old across seven sites in South Asia and sub-Saharan Africa. Several studies have reported that dehydrating rotaviral diarrhea still imposes a substantial disease burden among children younger than 5 years in both regions.^31^^,^^32^ The MAL-ED study (Malnutrition and Enteric Disease study) also demonstrated that rotavirus is the most common cause of dehydrating diarrhea in young children.^32^ In addition, the factors associated with rotavirus-positive MSD included age, gender, sociodemographic status, household wealth index, household drinking water source, handwashing practices, stunting, clinical features (fever, vomiting, IV rehydration), mother’s level of education, breastfeeding, and co-pathogens.

The most important finding in the study is a significant positive association between rotavirus-positive MSD and child growth in sub-Saharan Africa, even after adjusting for potential covariates. The association was consistent for two anthropometric parameters (LAZ/HAZ and WAZ). Other studies in Africa have also reported a positive relationship between rotaviral diarrhea and child growth.^20^^,^^21^ A cross-sectional study conducted in Uganda showed that rotaviral diarrhea was not significantly associated with nutritional status,^21^ and a study in Zambia observed that rotaviral diarrhea was more frequent among children with better nutritional status.^20^ Notably, along with other manmade influencing factors and climate factors, improved childhood nutrition and overnutrition may significantly contribute to the pathogenesis of symptomatic rotavirus infections.^23^ From a pathophysiologic perspective, rotavirus requires healthy epithelium for attachment and pathogenesis,^33^ and malnutrition affects the normal integrity of gut and immune system responses.^22^ In addition to natural infection, the preference of rotavirus for nutritionally intact intestinal epithelium may be a possible factor that explains the observed increases in anthropometric indices and the higher prevalence of rotavirus infection in sub-Saharan Africa. Age may be an additional factor that contributes to the higher reported incidence of rotaviral diarrhea in better nourished children,^34^ as younger children are less likely to be affected by the time-relative consequences of protein energy malnutrition, and the number of rotavirus receptor sites decreases with age, leading to lower susceptibility to infection.^34^ On the contrary, in South Asia, we observed that rotavirus-positive MSD was negatively associated with the wasting indicator (WLZ/WHZ) and was not associated with the other two parameters. Overall, rotaviral diarrhea affected the indices of nutritional status in children.

Many factors could explain the disparity in our findings between sub-Saharan Africa and South Asia. The mean baseline WLZ/WHZ, WAZ, and LAZ/HAZ were lower in South Asia than in sub-Saharan Africa. Studies have indicated that a lower WLZ/WHZ is a risk factor for rotaviral diarrhea and reported associations between rotavirus-positive MSD and indicator of wasting,^9^^,^^18^ the severity score,^18^ and the duration of diarrhea^18^; children who experienced rotaviral diarrhea for ≥6 days had a mean WLZ/WHZ score of −1.99, whereas children with <6 days’ duration had a mean WLZ/WHZ score of −1.43, and children with severe wasting had the highest severity.^18^ Wasting has also been observed as a risk factor for rotavirus infection in other studies.^35^ Although we did not access the severity score or duration in our study, the lower baseline WLZ/WHZ in South Asia than in sub-Saharan Africa may lead to diarrhea that is more severe and has a longer duration, which is more likely to lead to worsening of children’s nutritional status. Low WLZ/WHZ is often a result of acute significant food shortages and/or disease, low level of maternal education, too many pregnancies in a short period, and the involvement of mothers in activities within and reduce their ability to recover the children from the nutritional deficiency. An unmatched case-control study in Bangladesh reported that stunted and wasted children were more likely to experience dehydrating rotavirus-positive diarrhea than rotavirus-negative diarrhea.^9^ We also found that other co-pathogens, such as *Campylobacter, Giardia,* and ETEC, were less likely to be observed in children with rotavirus-positive MSD across the GEMS sites in Asia and Africa. Thus, co-pathogens had a negligible synergistic effect on nutritional changes due to rotavirus in this study. Thus, our findings reinforce the importance of focusing on rotavirus as the major cause of childhood diarrhea in developing countries.

In this study, young infants (0–11 months) were more likely to develop rotavirus-positive MSD than older children. Younger children are fond of crawling and commonly play on the ground, but they are less exposed to outdoor activities than older children. Our findings also support previous studies that reported infants (<12 months of age) are more vulnerable to rotavirus infection.^18^^,^^19^^,^^21^ This might be due to the weaker function of the immune system at a younger age or to a decrease in maternal antibodies after birth.^18^ Also, many young children are inappropriately provided with supplementary food along with breastfeeding at the age of 6 months, which frequently leads to malnutrition and diarrhea in lower-income countries.^18^ Among children with MSD presenting to health facilities involved in the GEMS, rotavirus-positive MSD was more common among girls than boys; however, at present, we do not have an explanation for this observation.

Fever, vomiting, and the requirement for IV rehydration are important factors associated with dehydrating diarrhea.^9^ The presence of fever, increased diarrheal episodes, frequent vomiting during the first 24 hours, and reduced consumption of ORS increases the severity of illness and may contribute to the development of MSD.^9^ Previous evidence reported that children admitted to hospitals with rotaviral diarrhea most often presented with diarrhea, vomiting, and unexplained fever.^36^ In our analysis, we also observed that rotavirus-positive MSD was significantly associated with vomiting, which can result in a reduced or improper ORS or other fluid intake at home. After a loss of serum bicarbonate, rotaviral diarrhea can lead to metabolic acidosis and subsequent vomiting.^36^ We also observed that rotavirus-positive MSD was significantly associated with the need for IV rehydration, but only in sub-Saharan Africa. There is also evidence that rotaviral infection causes secretory diarrhea by promoting fluid secretion as well as structural changes in the intestinal epithelium.^37^ A previous study found that adherence to WHO-recommended home-based management of diarrheal episodes, including adequate additional home fluids as well as continued feeding at home during diarrheal illness, was relatively low in sub-Saharan Africa^38^; this could better explain the increasing need for IV rehydration in sub-Saharan Africa compared with South Asia. Studies from Uganda^21^ and Ghana^35^ reported similar results, in that rotaviral diarrhea with vomiting and dehydration tends to be more serious than cases without these symptoms. A prospective study conducted in Burkina Faso indicated that children with rotavirus infection experienced fever in 42% of cases, vomiting in 82%, and severe dehydration in about 24%.^18^ Another study reported that rotavirus- or *E. coli*–infected children with smaller body sizes lost a greater proportion of body fluid per kilogram of body weight, which led to dehydration more often in this population than in children of normal weight.^39^

In our analysis, mothers’ level of education had no significant association with rotavirus-positive MSD. In contrast, other studies observed that children of mothers with a low level of education were more likely to have rotaviral diarrhea.^40^ Mothers are predominantly caretakers in developing countries. A low level of maternal literacy can prevent mothers from taking proper care of their children and may increase the risk of children being exposed to different childhood morbidities, including diarrhea and its consequences such as malnutrition.^9^ Moreover, in this study, we did not observe that breastfeeding had any significant protective effect against rotavirus-positive MSD children, and current breastfeeding was associated with a significantly higher risk of rotavirus-positive MSD compared with non-breastfeeding status. Although evidence suggests that breast milk confers protection against nonviral gastrointestinal pathogens, the protective effects of breast milk against viral pathogens appear weak.^41^ Breast milk provides essential elements such as human oligosaccharides, secretory immunoglobulin A, T lymphocytes, and B lymphocytes that play important roles in protecting infants from enteric pathogens,^42^ and components of breast milk have been associated with a decreased rate of diarrhea among children in both developed and developing countries.^43^^,^^44^ In a study in Burkina Faso, no protection against the rotavirus was observed in breastfed children; however, the prevalence of rotavirus was higher in breastfed children (67%, 6/9) than in those who were not breastfed (36%, 47/129).^18^ Similar evidence of a relationship between breastfeeding children and the rotavirus was also reported in Uganda.^21^ A review of several studies in Bangladesh inferred that breastfeeding makes a minor contribution to the prevention of rotavirus.^45^

In terms of water, sanitation, and hygiene (WASH), we found no association between sources of household drinking water, improved toilet facilities, and handwashing practices and rotavirus-positive MSD in sub-Saharan Africa, except for a negative association between handwashing before nursing children, perhaps because the caregivers mainly reported washing their hands with untreated water, not with soap. Similar findings from other studies in Africa indicate that WASH practice has no significant effect on children with MSD and rotavirus infection.^20^^,^^21^ In South Asia, we found that non-tube well water was associated with a higher risk of rotavirus-positive MSD and that primary caregivers washing their hands after cleaning children who defecated had a protective effect. Untreated or non-tube well water may get contaminated with a diverse range of enteric pathogens, which may result in frequent attacks of gastroenteritis and may lower children’s immune status and impaired nutritional status.^20^ Therefore, it is not implausible to presume that variations in one or more of the WASH factors linked to malnutrition may influence susceptibility to rotavirus in early childhood.

Our interpretation of these results also raises several limitations. The GEMS study gathered information only on children with MSD who visited the sentinel health center; thus, children without MSD, or who sought alternative care, or who did not report to the sentinel health center despite having MSD were not studied. Moreover, baseline weight was determined during discharge; however, some children were discharged before rehydration, which may have affected the baseline weight. The baseline weight for cases with MSD is likely to be affected by dehydration; thus, the positive effect of rotavirus-positive MSD on changes in weight from baseline to follow-up may be biased by the baseline weight of dehydrated children. In addition, the GEMS did not obtain any information related to maternal body mass index, prenatal intrauterine growth, postnatal variables, or blood markers related to nutritional status. Another limitation is that the effect of HIV and factors related to HIV associated with diarrhea and growth retardation could not be determined in this study.

The strengths of this study include the large unbiased sample size and high-quality standard laboratory analyses. However, irrespective of their sociodemographic or other circumstances, cost-free healthcare services were provided to all in this study. The remarkable feature of this study was a single follow-up visit roughly 60 days after enrollment, which allowed us to monitor growth outcomes of the children during vulnerable periods; hence, the observed association could be caused by acute infection.

## CONCLUSION

Our analysis showed that LAZ/HAZ declined in the 60–90-day follow-up period among children under 5 years with rotavirus-positive MSD in both sub-Saharan Africa and South Asia. In addition, after adjustments for demographic characteristics and co-pathogens, rotavirus-positive MSD infection was associated with lower WLZ/WHZ in South Asia but with a higher LAZ/HAZ and WAZ in sub-Saharan Africa. Our findings highlight the importance of nutritional interventions and WASH improvements to potentially reduce the burden and its sequel of rotaviral disease, such as stunting, during the first 5 years of life. Future prospective studies should be considered to explore diversity within and between the regions to accentuate the significance of policymaking for the prevention of rotaviral diarrhea and undernutrition. Moreover, given emerging evidence of their efficacy,^19^^,^^46^ we suggest that rotavirus vaccines be added to the WHO-recommended expanded program on immunization to minimize rotaviral diarrhea–related morbidity and mortality.

## Supplemental Materials

10.4269/ajtmh.23-0406 Supplemental Materials

## Data Availability

A publicly available GEMS dataset was analyzed in this study. These data can be obtained here: ClinEpiDB (https://clinepidb.org/ce/app/record/dataset/DS_841a9f5259).

## References

[1] Kosek M Bern C Guerrant RL, 2003. The global burden of diarrhoeal disease, as estimated from studies published between 1992 and 2000. Bull World Health Organ 81: 197–204. 12764516 PMC2572419

[2] UNICEF , 2019. *Child-Health/Diarrheal Disease*. Available at: https://data.unicef.org/topic/child-health/diarrhoeal-disease/. Accessed October 4, 2023.

[3] Kotloff KL , 2013. Burden and aetiology of diarrhoeal disease in infants and young children in developing countries (the Global Enteric Multicenter Study, GEMS): a prospective, case-control study. Lancet 382: 209–222. 23680352 10.1016/S0140-6736(13)60844-2

[4] ROTA Council , 2022. *The Epidemiology and Disease Burden of Rotavirus*. John Hopkins, International vaccine access center. Available at: https://preventrotavirus.org/wp-content/uploads/2022/04/ROTA-Brief3-Burden2022.pdf. Accessed October 4, 2023.

[5] GBD 2016 Diarrhoeal Disease Collaborators , 2018. Estimates of the global, regional, and national morbidity, mortality, and aetiologies of diarrhoea in 195 countries: a systematic analysis for the Global Burden of Disease Study 2016. Lancet Infect Dis 18: 1211–1228. 30243583 10.1016/S1473-3099(18)30362-1 PMC6202444

[6] Troeger C , 2018. Rotavirus vaccination and the global burden of rotavirus diarrhea among children younger than 5 years. JAMA Pediatr 172: 958–965. 30105384 10.1001/jamapediatrics.2018.1960 PMC6233802

[7] Mpabalwani M Oshitani H Kasolo F Mizuta K Luo N Matsubayashi N Bhat G Suzuki H Numazaki Y, 1995. Rotavirus gastro-enteritis in hospitalized children with acute diarrhoea in Zambia. Ann Trop Paediatr 15: 39–43. 7598436 10.1080/02724936.1995.11747747

[8] Odimayo MS Olanrewaju WI Omilabu SA Adegboro B, 2008. Prevalence of rotavirus-induced diarrhea among children under 5 years in Ilorin, Nigeria. J Trop Pediatr 54: 343–346. 18786984 10.1093/tropej/fmn081

[9] Yeasmin S Hasan SMT Chisti MJ Khan MA Faruque ASG Ahmed T, 2022. Factors associated with dehydrating rotavirus diarrhea in children under five in Bangladesh: an urban-rural comparison. PLoS One 17: e0273862. 36018895 10.1371/journal.pone.0273862 PMC9417038

[10] Chao DL Roose A Roh M Kotloff KL Proctor JL, 2019. The seasonality of diarrheal pathogens: a retrospective study of seven sites over three years. PLoS Negl Trop Dis 13: e0007211. 31415558 10.1371/journal.pntd.0007211 PMC6711541

[11] Yoon PW Black RE Moulton LH Becker S, 1997. The effect of malnutrition on the risk of diarrheal and respiratory mortality in children <2 y of age in Cebu, Philippines. Am J Clin Nutr 65: 1070–1077. 9094895 10.1093/ajcn/65.4.1070

[12] Hughes SM Amadi B Mwiya M Nkamba H Tomkins A Goldblatt D, 2009. Dendritic cell anergy results from endotoxemia in severe malnutrition. J Immunol 183: 2818–2826. 19625645 10.4049/jimmunol.0803518

[13] Rytter MJH Kolte L Briend A Friis H Christensen VB, 2014. The immune system in children with malnutrition – a systematic review. PLoS One 9: e105017. 25153531 10.1371/journal.pone.0105017 PMC4143239

[14] Vlasova AN , 2017. Protein malnutrition modifies innate immunity and gene expression by intestinal epithelial cells and human rotavirus infection in neonatal gnotobiotic pigs. mSphere 2: e00046-e17. 28261667 10.1128/mSphere.00046-17 PMC5332602

[15] Fischer DD , 2017. Protein malnutrition alters tryptophan and angiotensin-converting enzyme 2 homeostasis and adaptive immune responses in human rotavirus-infected gnotobiotic pigs with human infant fecal microbiota transplant. Clin Vaccine Immunol 24: e00172-e17. 28637803 10.1128/CVI.00172-17 PMC5583476

[16] Liu J Bolick D Kolling G Fu Z Guerrant R, 2016. Protein malnutrition impairs intestinal epithelial cell turnover, a potential mechanism of increased cryptosporidiosis in a murine model. Infect Immun 84: 3542–3549. 27736783 10.1128/IAI.00705-16 PMC5116730

[17] Iyer SS Chatraw JH Tan WG Wherry EJ Becker TC Ahmed R Kapasi ZF, 2012. Protein energy malnutrition impairs homeostatic proliferation of memory CD8 T cells. J Immunol 188: 77–84. 22116826 10.4049/jimmunol.1004027 PMC3244573

[18] Nitiema LW Nordgren J Ouermi D Dianou D Traore AS Svensson L Simpore J, 2011. Burden of rotavirus and other enteropathogens among children with diarrhea in Burkina Faso. Int J Infect Dis 15: e646–e652. 21763172 10.1016/j.ijid.2011.05.009

[19] Chissaque A , 2021. Rotavirus A infection in children under five years old with a double health problem: undernutrition and diarrhoea – a cross-sectional study in four provinces of Mozambique. BMC Infect Dis 21: 18. 33407207 10.1186/s12879-020-05718-9 PMC7788695

[20] Koyuncu A Simuyandi M Bosomprah S Chilengi R, 2020. Nutritional status, environmental enteric dysfunction, and prevalence of rotavirus diarrhoea among children in Zambia. PLoS One 15: e0240258. 33007035 10.1371/journal.pone.0240258 PMC7531814

[21] Nakawesi JS Wobudeya E Ndeezi G Mworozi EA Tumwine JK, 2010. Prevalence and factors associated with rotavirus infection among children admitted with acute diarrhea in Uganda. BMC Pediatr 10: 69. 20868488 10.1186/1471-2431-10-69 PMC2955671

[22] Verkerke H Sobuz S Ma JZ Petri SE Reichman D Qadri F Rahman M Haque R Petri WA Jr., 2016. Malnutrition is associated with protection from rotavirus diarrhea: evidence from a longitudinal birth cohort study in Bangladesh. J Clin Microbiol 54: 2568–2574. 27510830 10.1128/JCM.00916-16 PMC5035411

[23] Das SK , 2017. Long-term impact of changing childhood malnutrition on rotavirus diarrhoea: two decades of adjusted association with climate and socio-demographic factors from urban Bangladesh. PLoS One 12: e0179418. 28877163 10.1371/journal.pone.0179418 PMC5587254

[24] Kotloff KL , 2012. The Global Enteric Multicenter Study (GEMS) of diarrheal disease in infants and young children in developing countries: epidemiologic and clinical methods of the case/control study. Clin Infect Dis 55 *(* *Suppl 1* *):* S232–S245. 23169936 10.1093/cid/cis753 PMC3502307

[25] Kotloff KL , 2012. The Global Enteric Multicenter Study (GEMS) of diarrheal disease in infants and young children in developing countries: epidemiologic and clinical methods of the case/control study. Clin Infect Dis 55: S232–S245. 23169936 10.1093/cid/cis753 PMC3502307

[26] Panchalingam S , 2012. Diagnostic microbiologic methods in the GEMS-1 case/control study. Clin Infect Dis 55 *(* *Suppl 4* *):* S294–S302. 23169941 10.1093/cid/cis754 PMC3502308

[27] Wormser GP Hanna BA, 2004. Manual of Clinical Microbiology, 8th edition. Edited by Patrick R. Murray, Ellen Jo Baron, James H. Jorgensen, Michael A. Pfaller, and Robert H. Yolken Washington, D.C.: American Society for Microbiology Press, 2003. 2322 pp. $189.95 (cloth). Clin Infect Dis 38: 1199–1200.

[28] World Health Organization , 2006. WHO Child Growth Standards: Length/Height-for-Age, Weight-for-Age, Weight-for-Length, Weight-for-height and Body Mass Index-for-Age: Methods and Development. Geneva, Switzerland: WHO.

[29] De Onis M Onyango AW, 2008. WHO child growth standards. Lancet 371: 204. 18207015 10.1016/S0140-6736(08)60131-2

[30] World Health Organization , 2008. WHO Child Growth Standards: Training Course on Child Growth Assessment. Geneva, Switzerland: WHO.

[31] Omore R , 2016. Epidemiology, seasonality and factors associated with rotavirus infection among children with moderate-to-severe diarrhea in rural western Kenya, 2008–2012: the Global Enteric Multicenter Study (GEMS). PLoS One 11: e0160060. 27494517 10.1371/journal.pone.0160060 PMC4975461

[32] Mohan VR , 2017. Rotavirus infection and disease in a multisite birth cohort: results from the MAL-ED Study. J Infect Dis 216: 305–316. 28472348 10.1093/infdis/jix199 PMC5853665

[33] Ramig RF, 2004. Pathogenesis of intestinal and systemic rotavirus infection. J Virol 78: 10213–10220. 15367586 10.1128/JVI.78.19.10213-10220.2004 PMC516399

[34] Dewan N Faruque A Fuchs G, 1998. Nutritional status and diarrhoeal pathogen in hospitalized children in Bangladesh. Acta Paediatr 87: 627–630. 9686653 10.1080/080352598750014012

[35] Binka FN , 2003. Incidence and risk factors of paediatric rotavirus diarrhoea in northern Ghana. Trop Med Int Health 8: 840–846. 12950670 10.1046/j.1365-3156.2003.01097.x

[36] Bernstein DI, 2009. Rotavirus overview. Pediatr Infect Dis J 28: S50–S53. 19252423 10.1097/INF.0b013e3181967bee

[37] Thiagarajah JR Donowitz M Verkman AS, 2015. Secretory diarrhoea: mechanisms and emerging therapies. Nat Rev Gastroenterol Hepatol 12: 446–457. 26122478 10.1038/nrgastro.2015.111 PMC4786374

[38] Deichsel EL , 2023. Management of diarrhea in young children in sub-Saharan Africa: adherence to World Health Organization recommendations during the global enteric multisite study (2007–2011) and the Vaccine Impact of Diarrhea in Africa (VIDA) study (2015–2018). Clin Infect Dis 76: S23–S31. 37074440 10.1093/cid/ciac926 PMC10116557

[39] Black RE Merson MH Eusof A Huq I Pollard R, 1984. Nutritional status, body size and severity of diarrhoea associated with rotavirus or enterotoxigenic *Escherichia coli.* J Trop Med Hyg 87: 83–89. 6379203

[40] Dennehy PH , 2006. A case-control study to determine risk factors for hospitalization for rotavirus gastroenteritis in U.S. children. Pediatr Infect Dis J 25: 1123–1131. 17133157 10.1097/01.inf.0000243777.01375.5b

[41] Golding J Emmett PM Rogers IS, 1997. Gastroenteritis, diarrhoea and breast feeding. Early Hum Dev 49: S83–S103. 9363419 10.1016/s0378-3782(97)00055-8

[42] Morrow AL Ruiz-Palacios GM Altaye M Jiang X Guerrero ML Meinzen-Derr JK Farkas T Chaturvedi P Pickering LK Newburg DS, 2004. Human milk oligosaccharides are associated with protection against diarrhea in breast-fed infants. J Pediatr 145: 297–303. 15343178 10.1016/j.jpeds.2004.04.054

[43] Dewey KG Heinig MJ Nommsen-Rivers LA, 1995. Differences in morbidity between breast-fed and formula-fed infants. J Pediatr 126: 696–702. 7751991 10.1016/s0022-3476(95)70395-0

[44] Kramer MS , 2001. Promotion of Breastfeeding Intervention Trial (PROBIT): a randomized trial in the Republic of Belarus. JAMA 285: 413–420. 11242425 10.1001/jama.285.4.413

[45] Glass RI Stoll BJ, 1989. The protective effect of human milk against diarrhea: a review of studies from Bangladesh. Acta Paediatr Scand Suppl 351: 131–136. 2692384 10.1111/j.1651-2227.1989.tb11225.x

[46] Zeller M Rahman M Heylen E De Coster S De Vos S Arijs I Novo L Verstappen N Van Ranst M Matthijnssens J, 2010. Rotavirus incidence and genotype distribution before and after national rotavirus vaccine introduction in Belgium. Vaccine 28: 7507–7513. 20851085 10.1016/j.vaccine.2010.09.004

