# Supplementary material for: Impact of Rotaviral Diarrhea on Child Growth in Sub-Saharan Africa and South Asia in the Global Enteric Multicenter Study
Source: Am J Trop Med Hyg. 2024 Feb 20;110(4):749–58. doi: 10.4269/ajtmh.23-0406 (PMC10993829; doi:10.4269/ajtmh.23-0406)
Supplement: Supplemental Materials [file tpmd230406.SD1.pdf]

For HAZ

Model with interaction term

```
. logistic base_haz3 i.pn22 i.pn22##i.site1

Logistic regression                             Number of obs     =     22,544
                                                LR chi2(3)        =     168.04
                                                Prob > chi2       =     0.0000
Log likelihood = -13345.076                     Pseudo R2         =     0.0063
```

base_haz3	Odds Ratio	Std. Err.	z	P>|z|	[95% Conf.	Interval]
pn22						
Yes	.852336	.061489	-2.21	0.027	.7399523	.9817885
site1						
Africa	.6918662	.021566	-11.82	0.000	.6508629	.7354526
pn22#site1						
Yes# Africa	.9796573	.1001714	-0.20	0.841	.8017474	1.197046
_cons	.4845313	.0106015	-33.12	0.000	.464192	.5057617

```
Note: _cons estimates baseline odds.

.
. est store e1
```

Model without interaction term

```
. logistic base_haz3 i.pn22 i.site1

Logistic regression                             Number of obs     =     22,544
                                                LR chi2(2)        =     168.00
                                                Prob > chi2       =     0.0000
Log likelihood = -13345.096                     Pseudo R2         =     0.0063
```

base_haz3	Odds Ratio	Std. Err.	z	P>|z|	[95% Conf.	Interval]
pn22						
Yes	.8436428	.0431187	-3.33	0.001	.7632267	.9325319
site1						
Africa	.6905456	.0205004	-12.47	0.000	.6515121	.7319176
_cons	.4849875	.0103639	-33.86	0.000	.4650941	.5057317

```
Note: _cons estimates baseline odds.

.
. est store e2
```

```
. lrtest e1 e2

Likelihood-ratio test                                 LR chi2(1)  =      0.04
(Assumption: e2 nested in e1)                         Prob > chi2 =    0.8407
```

For WAZ

Model with interaction term

```
. logistic base_wazc i.pn22 i.pn22##i.site1
```

Logistic regression

Number of obs	=	22,553
LR chi2(3)	=	319.16
Prob > chi2	=	0.0000
Pseudo R2	=	0.0122

Log likelihood = -12904.925

base_wazc	Odds Ratio	Std. Err.	z	P>|z|	[95% Conf.	Interval]
pn22						
Yes	1.246639	.0854736	3.22	0.001	1.089882	1.425942
site1						
Africa	.5935876	.0190955	-16.21	0.000	.5573166	.6322191
pn22#site1						
Yes# Africa	.9274667	.0913435	-0.76	0.445	.7646558	1.124943
_cons	.4622598	.0101937	-34.99	0.000	.4427061	.4826771

Note: _cons estimates baseline odds.

```
.
. est store e1
```

Model without interaction term

```
. logistic base_wazc i.pn22 i.site1
```

Logistic regression

Number of obs	=	22,553
LR chi2(2)	=	318.58
Prob > chi2	=	0.0000
Pseudo R2	=	0.0122

Log likelihood = -12905.218

base_wazc	Odds Ratio	Std. Err.	z	P>|z|	[95% Conf.	Interval]
pn22						
Yes	1.201701	.0590484	3.74	0.000	1.091367	1.32319
site1						
Africa	.5888268	.0179041	-17.42	0.000	.5547605	.624985
_cons	.4640105	.0099649	-35.75	0.000	.4448849	.4839582

Note: _cons estimates baseline odds.

```
.
. est store e2
```

```
. lrtest e1 e2

Likelihood-ratio test                                 LR chi2(1)  =      0.59
(Assumption: e2 nested in e1)                         Prob > chi2 =    0.4442
```

For WHZ

Model with interaction term

```
. logistic base_whz3 i.pn22 i.pn22##i.site1
```

Logistic regression		Number of obs	=	22,525
		LR chi2(3)	=	42.34
		Prob > chi2	=	0.0000
Log likelihood = -9701.3633		Pseudo R2	=	0.0022

base_whz3	Odds Ratio	Std. Err.	z	P>|z|	[95% Conf.	Interval]
pn22						
Yes	1.450906	.118295	4.56	0.000	1.236629	1.702312
site1						
Africa	.9107856	.035816	-2.38	0.017	.8432246	.9837597
pn22#site1						
Yes# Africa	.9446536	.1065825	-0.50	0.614	.7572408	1.17845
_cons	.185791	.0052448	-59.62	0.000	.1757906	.1963603

Note: _cons estimates baseline odds.

```
.
. est store e1
```

Model without interaction term

```
. logistic base_whz3 i.pn22 i.site1
```

Logistic regression		Number of obs	=	22,525
		LR chi2(2)	=	42.09
		Prob > chi2	=	0.0000
Log likelihood = -9701.4906		Pseudo R2	=	0.0022

base_whz3	Odds Ratio	Std. Err.	z	P>|z|	[95% Conf.	Interval]
pn22						
Yes	1.408146	.0793466	6.07	0.000	1.260909	1.572575
site1						
Africa	.9045109	.0333397	-2.72	0.006	.8414708	.9722737
_cons	.1864533	.0050904	-61.52	0.000	.1767385	.1967021

Note: _cons estimates baseline odds.

```
.
. est store e2
```

```
. lrtest e1 e2
```

Likelihood-ratio test	LR chi2(1)	=	0.25
(Assumption: e2 nested in e1)	Prob > chi2	=	0.6139

Supplementary Files:

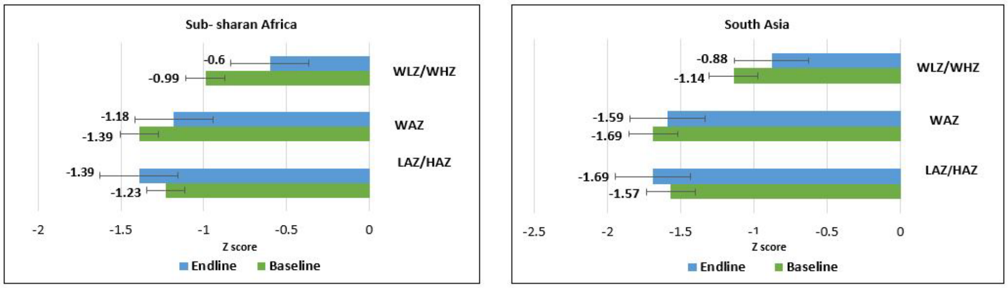

Fig S1: Mean baseline and endline height-for-age z score (LAZ/HAZ), weight-for-age z score (WAZ), and weight-for-height z score (WLZ/WHZ) among the rotavirus negative under 5 children from Sub-Saharan Africa and South Asia.
